# Transgenerational programming of longevity and reproduction by post-eclosion dietary manipulation in *Drosophila*

**DOI:** 10.18632/aging.100932

**Published:** 2016-03-28

**Authors:** Brian Xia, Steven de Belle

**Affiliations:** ^1^ Department of Biology, Dart Neuroscience LLC, San Diego, CA 92131, USA; ^2^ Canyon Crest Academy, San Diego, CA 92130, USA

**Keywords:** transgenerational inheritance, nutritional programming, longevity, reproduction, diet

## Abstract

Accumulating evidence suggests that early-life diet may program one's health status by causing permanent alternations in specific organs, tissues, or metabolic or homeostatic pathways, and such programming effects may propagate across generations through heritable epigenetic modifications. However, it remains uninvestigated whether postnatal dietary changes may program longevity across generations. To address this question of important biological and public health implications, newly-born flies (F0) were collected and subjected to various post-eclosion dietary manipulations (PDMs) with different protein-carbohydrate (i.e., LP, IP or HP for low-, intermediate- or high-protein) contents or a control diet (CD). Longevity and fecundity analyses were performed with these treated F0 flies and their F1, F2 and F3 offspring, while maintained on CD at all times. The LP and HP PDMs shortened longevity, while the IP PDM extended longevity significantly up to the F3 generation. Furthermore, the LP reduced while the IP PDM increased lifetime fecundity across the F0-F2 generations. Our observations establish the first animal model for studying transgenerational inheritance of nutritional programming of longevity, making it possible to investigate the underlying epigenetic mechanisms and identify gene targets for drug discovery in future studies.

## INTRODUCTION

Accumulating studies have established a strong link between early-life nutrition and adult health and disease [1-6]. In particular, maternal and postnatal malnutrition, among other environmental factors, can profoundly influence adult health outcomes and increase the subsequent risk of noncommunicable diseases (NCDs, including metabolic syndrome, diabetes, cardiovascular disease, etc.). The World Health Organization has recognized unhealthy diets as one of the leading causes of NCDs and such chronic diseases kill over 38 million people annually [7, 8]. Strikingly, 44% (or 16 million) of the deaths are premature, manifesting shortened average lifespan, one key characteristic of NCDs (cf. [9]). A very recent study, for example, indicates that combination of diabetes and heart disease may lead to a substantially lower life expectancy by over a decade [10]. The Organization has further recognized that maternal and child under-nutrition increases the subsequent risk of NCDs, accounts for 11% of the global burden of diseases, and is the underlying cause of 35% of early child death [11, 12].

It is now widely accepted that early-life nutrition programs the long-term health of an individual and his/her offspring [5, 6, 13, 14]. Such nutrition-mediated programming effects in fact have often been shown to be inheritable and transgenerational. Aiken and Ozanne recently performed a systematic and exhaustive search of the literature about transgenerational developmental programming for both epidemiological and animal studies [15]. Out of 45 primary research papers using rodent models that bred and examined a phenotype in offspring at least as far as the F2 generation, roughly 2/3 of them have employed early-life nutritional intervention (see Table 1 from [15]), highlighting “early-life nutrition” and “transgenerational effects” as key elements of developmental programming. However, the underlying mechanisms are only starting to emerge, with epigenetics as perhaps the most important mechanism through which diet and nutrition can directly influence the genome and response to diet itself [15-17]. Epigenetic modifications including DNA methylation, histone modifications, and non-coding RNA-based mechanisms are heritable but reversible changes that affect gene expression without altering the underlying DNA sequence. Such epigenetic “marks” influenced by early-life nutrition may therefore influence the subsequent health later in life and even across generations [5, 6, 15-19]. Importantly, trans-generational inheritance of nutritional programming of metabolic status has been recently demonstrated in flies [20], supporting “*Drosophila* as a valid model for studies of the epigenetic inheritance of metabolic state” [21] and the proof-of-concept of studying nutritional programming of other disease conditions in this much simpler while genetically tractable system.

**Table 1:** Statistical analyses for survival curves shown in Figure 1

Flies	Comparison	F0 parents: subjected to 7-day PDMs			F1 offspring			F2 offspring		
		Mantel-cox	Median lifespan		Mantel-cox	Median lifespan		Mantel-cox	Median lifespan	
		P value	Value	Change	P value	Value	Change	P value	Value	Change
**Virgin males**	LP vs. CD	< 0.0001	26 vs. 36	28% ↓	< 0.0001	30 vs. 40	25% ↓	< 0.0001	34 vs. 40	15% ↓
	IP vs. CD	< 0.0001	48 vs. 36	33% ↑	< 0.0001	50 vs. 40	25% ↑	0.0009	48 vs. 40	20% ↑
	HP vs. CD	0.61	34 vs. 36	6% ↔	0.85	38 vs. 40	5% ↔	0.67	40 vs. 40	0% ↔
**Virgin females**	LP vs. CD	< 0.0001	34 vs. 42	19% ↓	< 0.0001	35 vs. 46	24% ↓	< 0.0001	38 vs. 48	21% ↓
	IP vs. CD	< 0.0001	56 vs. 42	33% ↑	< 0.0001	56 vs. 46	22% ↑	< 0.0001	56 vs. 48	17% ↑
	HP vs. CD	0.034	40 vs. 42	5% ↓	0.016	42 vs. 46	9% ↓	0.0059	44 vs. 48	8% ↓
**Mated males**	LP vs. CD	< 0.0001	24 vs. 36	33% ↓	< 0.0001	30 vs. 36	17% ↓	< 0.0001	32 vs. 40	20% ↓
	IP vs. CD	< 0.0001	46 vs. 36	28% ↑	< 0.0001	48 vs. 36	33% ↑	< 0.0001	48 vs. 40	20% ↑
	HP vs. CD	0.47	35 vs. 36	3% ↔	0.44	38 vs. 36	6% ↔	0.21	38 vs. 40	5% ↔
**Mated females**	LP vs. CD	< 0.0001	32 vs. 40	20% ↓	< 0.0001	32 vs. 44	27% ↓	< 0.0001	36 vs. 46	22% ↓
	IP vs. CD	< 0.0001	52 vs. 40	30% ↑	< 0.0001	54 vs. 44	23% ↑	< 0.0001	54 vs. 46	17% ↑
	HP vs. CD	< 0.0001	36 vs. 40	10% ↓	0.0005	36 vs. 44	18% ↓	0.015	42 vs. 46	9% ↓

The Mantel-Cox (or log-rank) test was used to compare the survival distributions of LP-, IP-, or HP-treated F0 parents or their F1-F2 offspring with those of the control group (CD). The median lifespan data were also obtained to calculate the percentage changes in longevity, when compared with controls across the F0–F2 generations. ↓: significantly shortened lifespan; ↑: significantly improved lifespan; ↔: no significant lifespan change.

Several recent studies have also started to implicate early-life nutrition in the regulation of longevity [14, 19]. Flies had a shortened lifespan when raised on a high-sugar (HS) diet for 3 weeks [22]. Considering that the same HS diet elicited transgenerational metabolic alternations up to F2 offspring [20], the shortening effect of longevity may propagate across generations. Consistently, an earlier epidemiologic study demonstrated a negative correlation between the lifespan of grandsons and food consumption of parental grandfather during their slow growth period [23]. However, it remains unexplored whether immediate postnatal nutritional manipulations may program longevity, and whether such potential programming effects may propagate across generations.

*Drosophila* serves as a promising model for studying ageing and longevity, especially considering its short lifespan and usefulness for rapid gene discovery. A short lifespan makes it possible to complete one longevity experiment in about 3 months, rather than several years with a typical rodent model. Furthermore, *Drosophila* has been increasingly used for modeling human diseases and for drug discovery and development [24-26]. Importantly, Buescher et al. have employed a 7-day post-eclosion dietary treatment to demonstrate trans-generational metabolic programming in flies [20], and several diets have been used to show nutritional effects on learning and memory across multiple generations [27, 28]. Therefore, *Drosophila* presents itself as an excellent system to model transgenerational inheritance of nutritional programming of longevity and then to rapidly identify and characterize the underlying genetic and epigenetic pathways.

In this study, we examined whether specifically-defined PDMs may program longevity, and, if so, whether such programming effects may be long-lasting and inheritable across generations in *Drosophila*. The LP, IP and HP diets were adapted, because different protein-carbohydrate intakes appear to be essential for longevity in *Drosophila* [29, 30] and because similar diets have been used to demonstrate nutritional effects on learning and memory across multiple generations [27, 28]. We also assayed the effects of the same PDMs on lifetime fecundity, as reproduction is generally considered to trade off against longevity, with increased reproduction frequently associated with shortened lifespan [31]. We found that the IP diet improved longevity and lifetime fecundity while the LP and HP diets tended to decrease longevity and fecundity across three generations. Interestingly, no clear evidence was observed to support any trade-off between longevity and reproduction, suggestive of the feasibility of elevating both longevity and reproduction with proper nutrition across generations.

## RESULTS

### Transgenerational inheritance of nutritional programming of longevity

**Figure 1:**
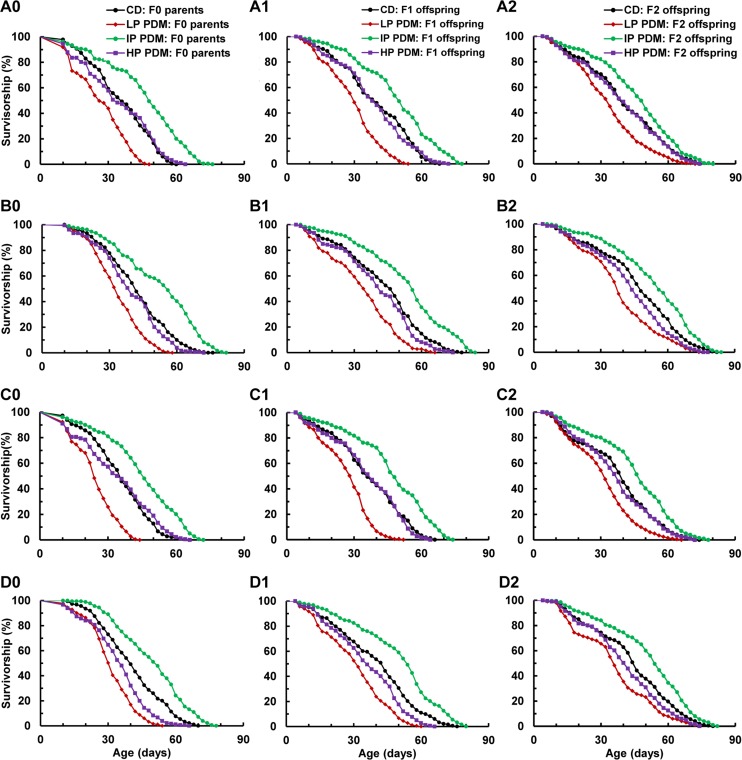
Survival curves of various flies across the F0–F2 generations after 7-day PDMs of the F0 parents with the LP, IP, or HP diet. (**A**) Virgin male (1st row), (**B**) virgin female (2nd row), (**C**) mated male (3rd row), and (**D**) mated female (4th row) flies; and (**0**) F0 parents (1st column), (**1**) F1 offspring (2nd column), and (**2**) F2 offspring (3rd column). N = 178–185 for the F0 and F1, and 199–205 for the F2 generation (see supplemental Table S2 for specific sample size for any given group).

Newly-born F0 flies were subjected to a 7-day PDM with one of three different diets (LP, IP and HP; see Table S1 for detailed description). Our longevity study was performed with these treated F0 flies and their F1, F2, and F3 progenies while being maintained on the CD food (i.e., without any additional exposure to the LP, IP or HP food across the F0–F3 generations) throughout their developmental and whole adult lives (see Figure S1 and Table S2 for detailed procedures and experimental design). The data were collected with 4 types of flies (i.e., virgin males and females, mated males and females) simultaneously. Such a design allowed us to assay whether the nutritional effects on longevity were similar among all 4 types of flies and further evaluate whether nutrition may affect longevity due to gender, mating, and/or reproduction. Overall, rather similar effects on longevity were observed for virgin males, virgin females, mated males, and mated females across the F0–F2 generations with the LP and IP diets but not with the HP diet (Figure 1 and Table 1).

**Table 2:** Statistical analyses of effects of gender, mating and reproduction on nutritional reprograming of longevity, using the same survival data as shown in Figure 1

Comparison	PDM diets	F0 parents: subjected to 7-day PDMs			F1 offspring			F2 offspring		
		Mantel-cox	Median lifespan		Mantel-cox	Median lifespan		Mantel-cox	Median lifespan	
		P value	Value	Change	P value	Value	Change	P value	Value	Change
**Virgin males vs. females**	CD	< 0.0001	36 vs. 42	14% ↓	0.0018	40 vs. 46	13% ↓	< 0.0001	40 vs. 48	17% ↓
	LP	< 0.0001	26 vs. 34	24% ↓	< 0.0001	30 vs. 35	14% ↑	0.0005	34 vs. 38	11% ↑
	IP	< 0.0001	48 vs. 56	14% ↑	0.0002	50 vs. 56	11% ↑	< 0.0001	48 vs. 56	14% ↑
	HP	0.023	34 vs. 40	15% ↑	0.14	38 vs. 42	10% ↔	0.07	40 vs. 44	9% ↔
**Mated vs. virgin males**	CD	0.41	36 vs. 36	0% ↔	0.066	36 vs. 40	10% ↔	0.08	40 vs. 40	0% ↔
	LP	0.0005	24 vs. 26	8% ↑	0.0064	30 vs. 30	0% ↔	0.062	32 vs. 34	6% ↔
	IP	0.15	46 vs. 48	4% ↔	0.081	48 vs. 50	4% ↔	0.45	48 vs. 48	0% ↔
	HP	0.97	35 vs. 34	3% ↔	0.061	38 vs. 38	0% ↔	0.071	38 vs. 40	5% ↔
**Mated vs. virgin females**	CD	0.31	40 vs. 42	5% ↔	0.078	44 vs. 46	4% ↔	0.11	46 vs. 48	4% ↔
	LP	0.015	32 vs. 34	6% ↑	0.023	32 vs. 35	9% ↑	0.31	36 vs. 38	5% ↔
	IP	0.0039	52 vs. 56	7% ↑	0.0066	54 vs. 56	4% ↑	0.11	54 vs. 56	4% ↔
	HP	< 0.0001	36 vs. 40	10% ↑	0.0006	36 vs. 42	14% ↑	0.15	42 vs. 44	5% ↔

The transgenerational longevity experiments were designed so that all 4 types of flies (i.e., virgin males and females, mated males and females) were assayed for longevity simultaneously. Such a design allowed us to ask whether nutrition may affect longevity differently because of gender, mating, and reproduction. For the potential effect of gender, survival curves and median lifespan for virgin males and females were compared within CD or each PDM diet across the F0–F2 generations (i.e., first 4 rows). For the potential effect of mating alone, survival curves and median lifespan for virgin and mated males were compared within CD or each PDM diet across the F0–F2 generations (i.e., next 4 rows). For the potential effect of reproduction (and mating), survival curves and median lifespan for virgin and mated females were compared within CD or each PDM diet across the F0–F2 generations (i.e., last 4 rows). ↑: significantly shortened lifespan; ↑: significantly improved lifespan; ↔: no significant lifespan change.

For the F0 generation, longevity was shortened after the LP PDM of the F0 parents for all 4 types of flies (Figure 1A0–D0, red diamonds; Table 1). Correspondingly, the median lifespan was decreased by 19–33% (Table 1). The HP PDM decreased longevity of the females significantly (P = 0.034 for virgin females and P < 0.0001 for mated females) or by 5–10% at the median lifespan, but did not affect longevity of males (P = 0.61 for virgin males and P = 0.47 for mated males; Figure 1A0–D0, purple squares; Table 1). In contrast, The IP PDM extended longevity for all 4 types of flies significantly, or by 28–33% at the median lifespan (Figure 1A0–D0, green circle; Table 1). These observations demonstrated that longevity was programmed after the 7-day PDMs in flies, and the HP treatment influenced longevity of the males and females differently.

For the F1 generation (Figure 1A1–D1), longevity was shortened after the LP PDM of the F0 parents for all 4 types of flies (red diamonds; P < 0.0001, Table 1). Consistently, the median lifespan was decreased by 17–27% (Table 1). The HP PDM decreased longevity of the females significantly (P = 0.016 for virgin females and P=0.0005 for mated females), or by 9–18 % at the median lifespan, but did not affect longevity of males (P = 0.85 for virgin males and P = 0.44 for mated males). In contrast, The IP PDM extended longevity for all 4 types of flies significantly (green circle; P < 0.0001, Table 1), or by 22–33% at the median lifespan. Considering that the germ cells (future gametes giving rise to F1 offspring) from the F0 males and females were also exposed to various PDMs while the F1 flies had never been exposed to LP, IP or HP diets, these data suggest that the nutritional programming of longevity was propagated from the F0 to F1 generation through parental effects.

For the F2 generation (Figure 1A2–D2), longevity was shortened after the LP PDM of the F0 parents for all 4 types of flies (red diamonds; P < 0.0001, Table 1), and the median lifespan was decreased by 15–22% (Table 1). The HP PDM decreased longevity of the females significantly (P = 0.0059 for virgin females and P=0.015 for mated females), or by 8–9 % at the median lifespan, but did not affect longevity of males (P = 0.67 for virgin males or P = 0.21 for mated males; Figure 1A2–D2, purple squares, and Table 1). The IP PDM again extended longevity for all 4 types of flies significantly (P = 0.0009 for virgin males and P < 0.0001 for the other three groups; Figure 1A2–D2 and Table 1), or by 17–20% at the median lifespan. Such observations clearly suggest that the nutritional programming of longevity may be transmitted to the F2 offspring through transgenerational inheritance, as both F1 (including their germ cells) and F2 flies have never been exposed to the LP, IP or HP diets. Such a conclusion was further supported by our observation of the F3 offspring (Figure S2) whereby longevity was still shortened after the LP PDM of F0 parents while improved after the IP PDM.

**Figure 2:**
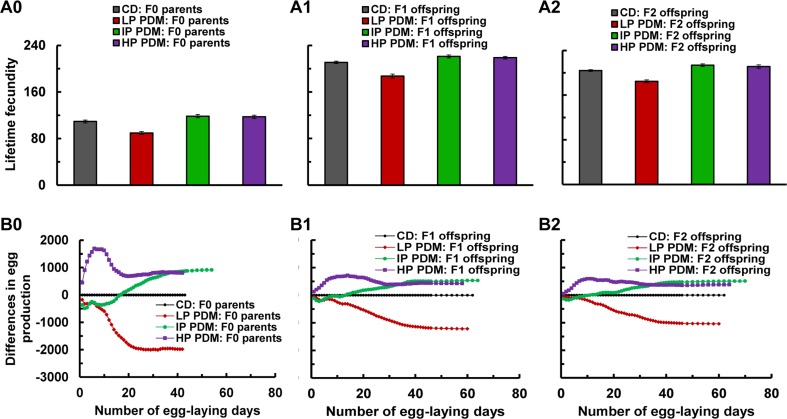
Lifetime fecundity and accumulative differences of egg production across the F0–F2 generations after 7-day PDMs of F0 parents with the LP, IP, or HP diet. (**A0–A2)** *Average lifetime fecundity* (i.e., total eggs produced by 1 female fly in its lifetime) was shown for the 100 mated females across three generations. The F0 females showed lower fecundity, as egg-production data collection was initiated when these flies were 11-day-old (because of the 7-day PDMs), while the F1 and F2 flies were 4-day-old. One-way ANOVA indicated a significant treatment effect among the control and 3 PDM groups in the F0 parents (P < 0.0001) and in their F1 and F2 offspring (P < 0.0001). Fisher's LSD (least significant difference) tests confirmed (P = 0.05) that lifetime fecundity was significantly reduced across all 3 generations after the LP PDM of the F0 flies, while increased across all 3 generations after the IP PDM or for the F0 and F1 generations after the HP PDM. However, lifetime fecundity for the F2 offspring was not increased after the HP PDM of the F0 flies (P > 0.05 for HP vs. CD). (**B0–B2**) *Accumulative differences of egg production* for the same 100 flies were plotted between CD and CD (black circle; 0 all the time in the graphs), LP (red diamond), IP (green circle), or HP (purple diamond) treatment over their whole “egg-laying” lives. The total eggs laid by the 100 control flies were 10933, 21081, and 20409 for the F0, F1, and F2 generations, respectively. The accumulative differences for the F1 and F2 generations were therefore normalized to the F0 generation (i.e., x 0.5186 or 10933/21081 for F1 and x 0.5357 or 10933/20409 for F2) for “straightforward” comparisons (but see supplemental Table S3 for details of egg-production results).

We also compared virgin males and virgin females within the CD or each PDM diet across 3 generations (Table 2, first 4 rows) to assess how gender affected longevity and transgenerational inheritance of longevity changes. Within the CD, virgin females lived about 15% longer than virgin males (14% for F0, 13% for F1, and 17% for F2 generations) at the median lifespan. This difference was maintained across the F0–F2 generations after both LP (P < 0.0001 for F0 and F1, P = 0.0005 for F2) and IP (P < 0.0001 for F0 and F2, P = 0.0002 for F1) PDMs of the F0 flies. Consistently, the median lifespan differences were 11–24% within the LP PDM and 11–14% within the IP PDM, suggesting that these PDMs affected males and females similarly. In contrast, such a difference was only maintained for the F0 generation for the HP PDM (P = 0.023, 15%). The F1 (P = 0.14) and F2 (P = 0.07) generations showed no significant difference, suggesting that the HP PDM affected males and females differently. Such analyses suggest that transgenerational inheritance of nutritional programming of longevity may be gender-independent, at least for the LP and IP PDMs, although female flies lived longer than males.

We next compared mated and virgin males within CD or each PDM diet across 3 generations (Table 2, 2nd 4 rows) to assess how mating affects longevity and nutritional programming of longevity changes. Mating did not produce a significant difference in lifespan between the mated and virgin males across F0-F2 generations for the control flies (P ≥ 0.066). Similarly, no significant difference was observed across the F0-F2 generations for the IP (P ≥ 0.081) and the HP (P ≥ 0.061) PDMs, suggesting that nutrition-induced longevity changes may be independent of mating for the IP and HP PDMs. A significant difference was present after the LP PDM between mated and virgin males across the F0–F1 generations (P ≤ 0.0064). However, such a difference was not maintained to the F2 generation (P = 0.062), supporting the notion that transgenerational longevity changes may be independent of mating for the LP PDM. Such analyses together suggest that transgenerational inheritance of nutritional programming of longevity may be independent of mating across all 3 PDMs, although the LP diet may affect the mating behavior of the F0 flies, and the effect appeared to propagate to the F1 generation likely through parental effects.

We finally compared virgin and mated females within CD or each PDM diet across 3 generations (Table 2, the last 4 rows) to assess how mating and reproduction affect longevity and nutritional programming effects of longevity. The mating and egg production within the CD medium did not produce a significant difference between mated and virgin females across the F0–F2 generations (P ≥ 0.078). In contrast, longevity was significantly shortened when comparing mated with virgin females for the LP, IP and HP diets across the F0 and F1 generations (P ≤ 0.023 for all comparisons). Correspondingly, the median lifespan decreased by 6–9 % for the LP PDM, 4–7% for the IP PDM, and 10–14% for the HP PDM. However, no difference was observed in the F2 generation for the LP, IP, and HP PDMs (P ≥ 0.11), suggesting that transgenerational longevity changes may be independent of mating and reproduction in the females. These analyses indicate that transgenerational inheritance of nutritional programming of longevity may be independent of mating and reproduction for all 3 PDMs, although all three PDMs may confound the mating and reproduction behavior of the F0 flies, and the effect appeared to propagate to the F1 generation likely through parental effects.

Taken altogether, our data demonstrate that nutritional programming of longevity may occur in the same F0 flies after PDMs with appropriate diets and then propagate to the F1 offspring through parental effects and further to the F2–F3 generations through transgenerational inheritance. The effects were very striking and appeared to be independent of gender, mating, and reproduction, at least for the LP and IP diets.

### Transgenerational inheritance of nutritional programming of reproduction

To assay whether the same PDMs may program reproduction of the F0 flies and their F1 and F2 progenies, 100 mated female flies were evenly divided into 10 subgroups, and their egg production was counted throughout their whole “egg-laying” lives (see Figure S1 and Table S3 for detailed procedures). The F0 females showed lower fecundity than their F1 and F2 offspring, as egg-production data collection was initiated when the flies were 11-days-old for the F0 (because of PDMs), while 4-days-old for the F1 and F2 generations.

For the F0 mated females, lifetime fecundity was greatly reduced after the LP PDM, while mildly increased after both IP and HP PDMs (Figure 2A0). The LP PDM appeared to decrease egg production early on (Figure 2B0, red diamond; see also Figure S3A0), and the trend continued as these flies died out faster than the “CD” controls. The IP PDM also decreased egg production early on, but the trend was reversed since these flies started to produce similar eggs per day while surviving better than the CD controls. In contrast, the HP PDM greatly increased egg production early on (Figure 2B0 and S3A0, purple circles), and the differences remained over time. Consequently, the 100 “LP” F0 flies laid 1984 fewer eggs, while the “IP” and “HP” flies 913 and 802 more eggs, as compared to the “CD” F0 flies (Figure 2B0). These observations indicate that the same PDMs programmed reproduction of the F0 parents.

**Figure 3:**
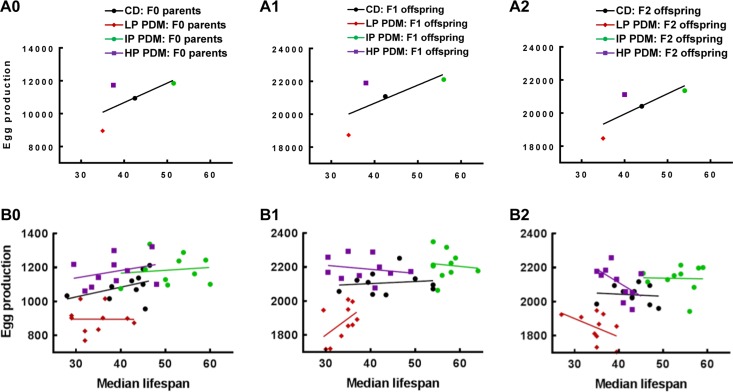
No evidence for trade-off between longevity and reproduction. (**A0–A2**) Correlation analyses, using total egg production and median lifespan of the 100 flies from the control (CD) and three PDMs, indicated that longevity and reproduction were positively correlated across the F0-F2 generations (r = 0.64, 0.68, and 0.75 for F0, F1, and F2). (**B0–B2**) Correlation analyses, using total egg production and median lifespan of the 10 subgroups (10 flies per group) within each dietary treatment, again indicated no obvious negative correlation across four dietary treatments and three generations (−0.01 ≤ r ≤ 0.41 for F0, −0.21 ≤ r ≤ 0.50 for F1, and −0.55 ≤ r ≤ −0.03 for F2).

Similar to that of the F0 generation, lifetime fecundity of the F1 offspring (Figure 2A1) was significantly reduced after the LP PDM, while mildly improved after the IP and HP PDMs of the F0 parents. As we observed for the F0 generation, the “LP” F1 flies produced fewer eggs early on, and the trend continued throughout their whole “egg-laying” lifetime; The “IP” F1 flies produced fewer eggs early on, but the trend was reversed over time; in contrast, the “HP” F1 flies showed increased egg production early on, and the differences remained over time (Figure 2B1 and S3A1). Accumulatively, the 100 F1 “LP” flies laid 1218 fewer eggs, while the F1 “IP” and “HP” flies 533 and 422 more eggs (after “normalization”, see Figure 2 legend for details). These observations indicate that the nutritional programming of reproduction propagated from the F0 flies to the F1 progeny through parental effects, as the F1 flies have never been exposed to the LP, IP, or HP diets.

Similar to that of the F0 and F1 generations, lifetime fecundity of the F2 offspring (Figure 2A2) was significantly reduced after the LP PDM of the F0 flies, while increased after the IP PDM of F0 flies. However, lifetime fecundity for the F2 offspring was not increased after the HP PDM of the F0 flies (P>0.05 for HP vs. CD). Again as for the F0 and F1 generations, we found that the “LP” F2 flies produced fewer eggs early on, and the trend continued throughout their whole “egg-laying” lifetimes; and the “IP” F1 flies produced fewer eggs early on, but the trend was reversed. In contrast, “HP” F1 flies showed increased egg production early on, and the differences remained over time (Figure 2B2 and S3A2). Accumulatively, the 100 F2 “LP” flies laid 1040 fewer eggs, while the F2 “IP” and “HP” flies 509 and 378 more eggs (after “normalization”). Such observations demonstrate that the nutritional programming of reproduction may be transmitted to the F2 offspring through transgenerational inheritance, as both the F1 and F2 flies have never been exposed to LP, IP or HP diets.

The differences in lifetime fecundity and total egg production between the “CD” and various “PDM” flies across the F0–F2 generations suggest that the nutritional programming of reproduction (1) was induced after PDMs of the F0 flies; (2) might be transmitted from the F0 to F1 generation through parental effects; and (3) further transmitted at least to F2 offspring through transgenerational inheritance.

### No evidence for trade-off between longevity and reproduction

Reproduction is a costly process and generally considered to trade off against longevity, with increased reproduction frequently associated with shortening lifespan [31]. Longevity data were also collected for the 100 (10 × 10 groups) females during the reproduction experiments, allowing us to examine whether there may be any trade-off between longevity and reproduction. Correlation analyses, using total egg production and the median lifespan of the 100 flies, indicated that longevity and reproduction were in fact positively correlated across the F0-F2 generations (Figure 3A0–A2).

Correlation analyses, using total egg production and the median lifespan of the 10 subgroups (10 flies per group) within each dietary treatment, showed no clear evidence for any trade-off (i.e., negative correlation) between longevity and reproduction for the CD or 3 PDM diets and across the F0-F2 generations (Figure 3B0–B2). Taken together, these data, with 4 different dietary treatments and across 3 generations of flies, showed no evidence for any trade-off between longevity and reproduction. Therefore, these lab-raised flies may have evolved to “abandon” such trade-off constraints under abundant food supplies through hundreds of generations, supporting the feasibility of improving both longevity and reproduction with proper nutrition across generations.

## DISCUSSION

Our observations demonstrate that the nutritional programming of longevity may occur in the same F0 flies after PDMs with multiple diets and then propagate to the F1 offspring through parental effects and further to the F2–F3 offspring through transgenerational inheritance. The programming effects were obvious and appeared to be independent of gender, mating, and reproduction (at least for the LP and IP diets). Interestingly, no clear evidence was observed for any potential trade-off between longevity and reproduction, suggestive of the feasibility of improving both longevity and reproduction with proper nutrition across generations. Altogether, these findings establish the first animal model to study transgenerational nutritional programming of longevity through early-life experience, and would facilitate investigations to identify the underlying molecular and epigenetic mechanisms and eventually translational research to combat the burden of NCDs and extend human health and longevity through optimizing the early-life nutritional environment.

Postnatal nutritional programming has been much less studied, as compared to prenatal or fetal programming. Adjustment of litter size (e.g., large litter for undernourishment or small litter for overnourishment), modification of milk formula (e.g., carbohydrate-enriched), and lactational environment in the dam (e.g., maternal calories or protein restriction, maternal diabetes or obesity) have been used for altered nutritional experiences in the immediate postnatal life of rodents [15, 32, 33]. Many of the programming effects appear to be long-lasting and persist into adulthood, but transgenerational inheritance has not been formally explored in this context (cf. [15, 32]). Nevertheless, epigenetic regulations have been revealed for some postnatal nutritional treatments, such as early postnatal exposure to the high-carbohydrate or high-fat diets [34, 35], suggestive of involvement of transgenerational inheritance. Consistently, certain aspects (i.e., increased glucose and trehalose levels in larval stages) of the programmed metabolic status after a 7-day post-eclosion feeding of an HS diet to the female flies persist and propagate through the F2 generations, demonstrating that *Drosophila* display transgenerational inheritance of metabolic state after altered immediate post-eclosion nutritional experience [20].

It is worth noting that the above-mentioned transgenerational effect of metabolic status to the F2 generation represents a true cross-generation transmission, rather than a direct consequence of the original HS dietary feeding [20]. Here, virgin female flies (F0), after being subjected to an HS diet for 7 days, are transferred to a low-sugar (LS) control diet for mating with untreated male flies, and the F1 virgin females are then cultured on the LS food for 7 days prior to mating to generate the F2 population which are also raised on the LS diet. The germ cells (future gametes) from the F0 females therefore would also be directly exposed to the HS dietary influence, while those from the F1 females would not. As the F2 flies are generated from F1 germ cells never exposed to the HS diet, any displayed programming effects must result from transgenerational inheritance or transmission.

Such a paradigm was thus adapted for our study, with a few important modifications that may potentially improve the transgenerational inheritance effects. First, virgin flies (including both females and males) were collected within 4 hours after eclosion — in fact usually 2-3 hours for most virgin collections; each collection was quickly divided into 4 equal groups and then placed immediately on the LP, IP, HP, or CD for 7 days. Instead, virgin females were subjected to the HS diet within 24 hours of eclosion previously [20]. As in most mammalian species, neural and organ development in *Drosophila* is not complete at eclosion (birth) and continues in days (the immediate post-eclosion period). Fiber number in the adult mushroom bodies (MB; sensory integration centers of the insect brain), for example, markedly increases during the first week after eclosion [36]. Glial cell outgrowth also appears at this time, with its resulting processes forming a mesh-like network inside the MB *03b1*β lobes within 10 days of eclosion [37]. The lamina grows in volume during the first days after eclosion, and it grows more in the light than in darkness. The lamina is similarly small when flies are kept in the dark only for the first 12 hours of their adult lives or raised for 4 days in constant darkness, highlighting that the first day of adulthood appears to be particularly critical for development [37, 38]. Furthermore, epigenetic programming continues with significant dynamism across the early post-eclosion period (cf. [39]). With the consideration that greatest sensitivity may occur during the period of most rapid growth and maturation, a dietary treatment delivered within the shortest possible time after eclosion may induce the maximal long-lasting programming effects. Developmental plasticity during the immediate post-eclosion period then affords the offspring the adaptive ability to respond to an altered nutritional environment [38, 40, 41].

The second important modification was that both newly-born males and females were subjected to the immediate PDMs, instead of only the females as in Buescher et al. [20], or either males or females as in most rodent studies (reviewed in [15]). The majority of rodent studies have focused on the maternal transmission to demonstrate transgenerational programming effects of early-life nutritional experience [15]. Although much less studied, the transgenerational inheritance effects through the paternal transmission have recently been established for paternal over-nutrition, undernutrition, high-fat diet, and low-protein diet [42-46]. In particular, transgenerational glucose intolerance, induced by intrauterine hyperglycemia or by in utero undernutrition in mice, may be transmitted via either the maternal or paternal line while potentially through different mechanisms [45, 46], suggesting that the transgenerational nutritional programming effects may potentially be additive when induced in both father and mother. Therefore, our design would allow us to induce potentially the largest possible alternation of longevity and reproduction across generations, with potential contributions from both treated males and females. The limitation was in not being able to distinguish potentially different contributions from males and females, something that we did not intend to address in this study.

The third important consideration was that several food diets from other literature were used, with recipes slightly modified to produce isocaloric food media at various protein-carbohydrate contents different from that of CD, a food medium routinely used in our laboratory (see methods and Supplemental Table S1 for details). Our main considerations were that (1) these diets are either widely used across the fly community or used elsewhere to examine cross-generation effects on fly behavior [27, 28], (2) they contain a wide range of protein and carbohydrate contents, very different from CD, and (3) our flies have been adapted to the CD food over hundreds of generations, justifying the use of CD as the control diet. In contrast, using the LS and HS diets may cause several complications. These two diets are semi-defined food media, very different from our CD or any other routinely-used medium. Considering that chemically-defined food media affect longevity differently from routinely used ones across a wide range of protein-carbohydrate contents [29, 30], combined use of the LS and HS diets with our CD may be problematic. Importantly, the HS diet has a protein-carbohydrate content close to that of CD ([20], Supplemental Table S1), and the caloric value is dramatically different for the LS and HS diets [20]. These two semi-defined food media were therefore not used in our study.

Using this modified PDM paradigm, longevity was assayed with the PDM-treated F0 flies and their F1–F3 offspring (Figure 1). The PDM with the LP diet shortened longevity of all 4 types of flies (virgin males and females, mated males and females) across the treated F0 flies to the F3 generation. The PDM with the IP diet extended longevity of all 4 types of flies across the treated F0 flies to the F3 generation. The PDM with the HP diet shortened longevity of the virgin and mated females (while not males) across the treated F0 flies to the F2 generation (but not the F3 offspring). As discussed above, the nutritional programming effects, induced in the PDM-treated F0 flies and propagated to the F1 generation, would reflect parental effects, as the germ cells from the F0 flies were under influence of these PDMs; the effects propagated from the F1 to F2 generation represented a transgenerational transmission or inheritance, as the germ cells from the F1 offspring were not exposed to LP, IP, or HP diets. Taken together, our data demonstrate that the nutritional programming of longevity occurred in the same F0 flies after PDMs with several diets and was then propagated to the F1 offspring through parental effects and further to the F2–F3 offspring through transgenerational inheritance (Figure 1).

Interestingly, the transgenerational programming effect was similar among all 4 types of flies within each PDM for the LP and IP diets. Further data analysis confirmed that the transgenerational effect to the F2 generation was independent of gender, mating, and egg reproduction after PDMs of the F0 flies with the LP and IP diets (Table 2). Our PDM paradigm, with both newly-born males and females receiving the same PDMs simultaneously before mating, prevented us from distinguishing the potentially different contributions from the treated males and females to their offspring, and thus determining the existence of sexual dimorphism in the transgenerational inheritance of nutrition-induced longevity programming for the LP and IP diets. In contrast, the PDM with the HP diet induced the transgenerational programming effect only in virgin and mated females, but not in virgin and mated males of the F2 offspring, supporting the existence of sexual dimorphism, at least for this particular HP diet. Consistently, sexual dimorphism is present in the transgenerational inheritance of certain metabolic syndromes in rodent studies [45, 46].

The differences in lifetime fecundity and total egg production between the “CD” and “PDM” flies across the F0–F2 generations revealed the nutritional programming of reproduction and its transgenerational inheritance at least to the F2 generation. The effects were generally mild for all three diets used (Figure 2). Nevertheless, these data allowed us to perform correlation analyses between reproduction and longevity, and thus to conclude that there may not be a trade-off between longevity and reproduction. The no-trade-off observation strengthened the idea that transgenerational nutritional programming of longevity may be independent of mating and reproduction, and supported the feasibility of elevating both longevity and reproduction with proper nutrition across generations.

Our observations about transgenerational nutritional programming of longevity (Figure 1, Table 1), which especially may be independent of gender, mating, and reproduction at least for 2 out of 3 diets used (Figure 1; Table 2), thus establish the first animal model to study this newly-recognized phenomenon [19]. As recently surveyed, there is no such reported study yet on transgenerational inheritance of programmed longevity from early-life nutritional experience or any other developmental programming interventions [15, 19]. Nevertheless, with a strong link between early-life nutrition and the long-term health of an individual and his/her offspring well established (cf. [1, 3, 5, 6, 15]), recent studies have implicated that early-life nutrition may program longevity across generations [19]. Our results have confirmed this implication and, more generally, have established an animal model system for further studies.

This model offers several tractable advantages, in particular to identify and characterize the epigenetic mechanisms underlying the nutrition-mediated cross-generation programming of longevity and NCDs. The relatively short rearing time and lifespan of *Drosophila* facilitate longevity experiments over multiple generations in a reasonable time scale (this study). In addition, various dietary (e.g., particular nutrient-depleted or enriched, semi- or chemically-defined) manipulations and well-conserved (e.g., insulin/IGF, TOR and sirtuin) signaling pathways have been described and characterized for studies of longevity and aging-related diseases (e.g., obesity, cardiomyopathy and memory disability) in flies [20, 22, 27, 29, 30, 47-55]. These have been necessary and critical for rapid identification and characterization of any epigenetic mechanisms. Practical dissection of various tissues and diverse choices of genetic manipulations may also be readily applied to explore the relationship among diet, corresponding disease and underlying epigenetic mechanism (cf. [22, 47, 56]). Furthermore, major epigenetic mechanisms (e.g., DNA methylation, histone modifications and non-coding RNA interference) are present in the model system [57], with clear evidence for histone modifications (e.g., methylation, acetylation and biotinylation; [47, 58-61]) and at least 2 microRNAs [62, 63] participating in the regulation of longevity. However, there is no report yet implicating DNA methylation in longevity regulation, because it was long believed to be absent in adult flies (cf. [64]). Importantly, recent studies have reported convincing support for the existence of low-level of DNA methylation in adult *Drosophila* [65, 66], and an active yet-to-be-identified DNA methyltransferase, with sequence specificity confirmed by the presence of asymmetric methylation at corresponding sites in the genomic DNA [66]. Considering that transgenerational programming through DNA methylation has been documented in rodents (cf. [15, 42, 67], it would be likely that early life dietary manipulations may program longevity by influencing DNA methylation in flies. Finally, our study and the earlier one, using the same 7-day post-eclosion dietary treatment to demonstrate nutrition-mediated metabolic programming up to the F2 generation [20], support the use of post-eclosion adult stage to assay the epigenetic mechanisms underlying transgenerational programming of longevity or aging-related diseases in *Drosophila*.

We expect that our model system would facilitate studies to identify the underlying molecular and epigenetic mechanisms and eventually translational research to combat the burden of NCDs and extend the human health and longevity through optimizing the early-life nutritional environment. Such longevity studies may also reveal a common mechanism for preventing many NCDs. In fact, the link between early-life nutrition and adult health and disease has gradually been recognized as a cornerstone of public health nutrition programs globally. The World Health Organization recently published global targets and a comprehensive implementation plan for the nutrition of mothers, infants, and young children, aiming to alleviate the double burden of malnutrition in children, starting from the earliest stages of development [11]. Noticeably, *Drosophila* may also be easily adapted to model various exposures such as stress or environmental contaminants/toxicants [27, 68] with similar treatment paradigms and thus to help address the growing issue of food security related to industrialization and globalization [69, 70].

## METHODS

### Flies

Wild-type isogenic w^1118^ strain (stock #5905, Bloomington Stock Center) was used throughout the study. All the flies were maintained in Forma incubators with controlled temperature (25°C) and humidity (40%) on a 12:12 light-dark cycle (with light on at 8am).

### Diet

The food recipes, along with the calorie, protein, and carbohydrate information for the control diet (CD) and three other diets used for PDMs of the F0 parents, were provided in Supplemental Table S1. CD is a food medium routinely used in our laboratory, containing ~8.5% protein and ~76.5% carbohydrate. The “LP” (Low Protein) diet was adapted from Xia et al [28], a food medium which contains much less protein (~3.3%) while much more carbohydrate (~90.5%), and abolishes learning and memory across generations. The “IP” (Intermediate Protein) diet was adapted from the “Beijing Diet” described by Guo et al [27], a food medium containing less protein (~5.5%) while more carbohydrate (~87.5%). The “HP” (High Protein) diet was adapted from a widely used “standard diet” as described at Bloomington Stock Center (http://flystocks.bio.indiana.edu/Fly_Work/media-recipes/bloomfood.htm), a food medium containing increased protein (~13.5%) while less carbohydrate (~69.5%). This diet has been extensively used across the fly community. All three diet recipes were slightly modified to be isocaloric (0.77 calories/gm food; Supplemental Table S1). All flies had free access to abundant food, and were expected to eat similar amounts of food with the same amount of calories, making it an unlikely explanation that the observed longevity changes were due to potentially different caloric intakes.

### Post-eclosion dietary manipulation and experimental design (see Supplemental Figure S1 for details)

Virgin males and females were collected within 4 hours after eclosion and then maintained on LP, IP, HP, or CD for 7 days (i.e., STEP 1), a treatment protocol adapted from Buescher et al. in which a 7-day post-eclosion dietary treatment of virgin females only was employed to demonstrate transgenerational metabolic programming in *Drosophila* [20]. Both newly-born males and females were treated, instead of only either males or females as in most of literature (cf. [15, 20]). This design enabled the induction of potentially large changes in longevity and fecundity across generations, with contributions from both treated males and females. However, it did not allow us to distinguish the potentially different contributions of males and females to their offspring. We did not intend to address this in our study.

Three groups of about 60 virgin males and females were then transferred to CD for 3 days after 7-day PDM with LP, IP or HP diet, while the others (8 groups) were mated with each other for 3 days, while being kept on CD (i.e., STEP 2). Then in STEP 3, (1) 3 groups (~180 flies) of virgin males, virgin females, mated males, and mated females were used for longevity analyses; (2) 100 mated females were evenly split into 10 subgroups (10x 10) and used for both egg production and longevity analyses; and (3) 180 mated females were split into 4 groups and used for generating the F1 offspring while being maintained on CD all the time.

Similar analyses were implemented with the F1 and F2 offspring, by repeating STEPs 2-3 (i.e., without PDMs from STEP 1) as for the F0 parents while using newly-born virgin males and females, except that roughly 4x 50 flies were used for longevity analyses of the F2 offspring. For F3 offspring, virgin F3 males and females were collected within 4 hours after eclosion, and only 4 groups of 50 mated females were used for longevity analyses (Supplemental Figure S2).

### Longevity assay

All data were collected in a blind and balanced manner, with different groups of flies blind-coded and balanced for various sources of variation, including (1) number of flies in each vial and for each PDM, (2) food level across vials, and (3) light exposure, humidity, and temperature by regular rotation through fixed locations in incubators. Then, a large number of flies (i.e., ~ 3x 60 or 180 for F0 and F1 generations and 4x 50 or 200 for F2 and F3 generations) were used to ensure systematic and sufficient data collection, and reproducibility (see Supplemental Table S2 for details). Flies were transferred onto new CD vials every 2 days, ensuring that all flies had access to fresh food, and the feeding environment remained fresh and consistent. The date and number of dead flies for each vial were recorded at the time when the flies were being transferred. All dead flies were carefully removed with a spatula. Any fly that accidentally escaped or died would not be considered. Longevity data were also collected for the 100 mated females used for egg production analyses. Similar effects were observed for each PDM diet on longevity across the F0–F2 generations (data not shown), supporting reproducibility.

The F0 parent generation was first subjected to the 7-day PDMs with various diets, and then a 3-day “maintenance” (for virgin males and females) or “mating” (for mated males and females) period. The subsequent F1-F3 generations were never exposed to the 3 PDM diets, but still went through the 3-day maintenance or mating period before longevity analyses. Therefore, the longevity data were collected from 11-day-old for F0 parents, and 4-day-old flies for subsequent F1-F3 offspring (as shown in Figure 1).

### Reproduction assay

All data were collected in a blind and balanced manner, similar to that of longevity analyses. Then 10 subgroups of 10 flies were used to ensure systematic and sufficient data collection, and reproducibility. Flies were transferred onto new CD vials every day for the first 40–45 days and then every other day (when very few eggs were laid), ensuring that all flies had access to fresh food, and the feeding environment remained fresh and consistent. Both eggs and eggshells (if any; larvae were ignored) were counted under microscope and recorded for each vial. Dead flies were also counted and recorded for longevity analyses, allowing us to confirm reproducibility of longevity analyses (data not shown) and evaluate the potential trade-off effect between longevity and reproduction (Figure 3).

### Data analysis

All longevity analyses were run through GraphPad Prism. Prism uses the Mantel-Cox test to generate survival curves and compares the survival distributions of two samples to determine the significance of any changes. The median lifespan data were also obtained to calculate the percentage changes of the longevity. The transgenerational longevity experiments were designed so that all 4 types of flies (i.e., virgin males and females, mated males and females) were assayed for longevity simultaneously. Such a design allowed us to ask whether nutrition may affect longevity differently because of gender, mating, and/or reproduction (see Table 2 for details).

For lifetime fecundity, one-way analysis of variance (ANOVA), followed with post-hoc Fisher's least significant difference (LSD), was used to determine the significance between each PDM diet and CD (see Figure 2 for details).

Correlation analyses, using total egg production and the median lifespan of the same flies, were performed to determine whether reproduction and longevity may be correlated (see Figure 3 for details).

## SUPPLEMENTARY FIGURES AND TABLES



## References

[1] Barker DJ (2004). Developmental origins of adult health and disease. J Epidemiol Community Health.

[2] Barker DJ, Osmond C, Golding J, Kuh D, Wadsworth ME (1989). Growth in utero, blood pressure in childhood and adult life, and mortality from cardiovascular disease. BMJ.

[3] Gluckman PD, Hanson M (2006). Developmental Origins of Health and Disease.

[4] Hales CN, Barker DJ, Clark PM, Cox LJ, Fall C, Osmond C, Winter PD (1991). Fetal and infant growth and impaired glucose tolerance at age 64. BMJ.

[5] Langley-Evans SC (2015). Nutrition in early life and the programming of adult disease: a review. J Hum Nutr Diet.

[6] Vickers MH (2014). Early life nutrition, epigenetics and programming of later life disease. Nutrients.

[7] World Health Organization (2011). Global Status Report on Noncommunicable Diseases 2010. http://http://apps.who.int/iris/bitstream/10665/44579/1/9789240686458_eng.pdf.

[8] World Health Organization (2014). Global Status Report on Noncommunicable Diseases. http://apps.who.int/iris/bitstream/10665/148114/1/9789241564854_eng.pdf?ua=1.

[9] Taguchi A, White MF (2008). Insulin-like signaling, nutrient homeostasis, and life span. Annu Rev Physiol.

[10] Di Angelantonio E, Kaptoge S, Wormser D, Willeit P, Butterworth AS, Bansal N, O'Keeffe LM, Gao P, Wood AM, Burgess S, Freitag DF, Pennells L (2015). Association of Cardiometabolic Multimorbidity With Mortality. JAMA.

[11] World Health Organization (2014). Comprehensive Implementation Plan on Maternal, Infant and young Child Nutrition. http://apps.who.int/iris/bitstream/10665/113048/1/WHO_NMH_NHD_14.1_eng.pdf?ua=1.

[12] Black RE, Allen LH, Bhutta ZA, Caulfield LE, de Onis M, Ezzati M, Mathers C, Rivera J (2008). Maternal and child undernutrition: global and regional exposures and health consequences. Lancet.

[13] Soubry A (2015). Epigenetic inheritance and evolution: A paternal perspective on dietary influences. Prog Biophys Mol Biol.

[14] Tarry-Adkins JL, Ozanne SE (2011). Mechanisms of early life programming: current knowledge and future directions. Am J Clin Nutr.

[15] Aiken CE, Ozanne SE (2014). Transgenerational developmental programming. Hum Reprod Update.

[16] Haggarty P (2013). Epigenetic consequences of a changing human diet. Proc Nutr Soc.

[17] Vickers MH (2014). Developmental programming and transgenerational transmission of obesity. Ann Nutr Metab.

[18] Hochberg Z, Feil R, Constancia M, Fraga M, Junien C, Carel JC, Boileau P, Le Bouc Y, Deal CL, Lillycrop K, Scharfmann R, Sheppard A, Skinner M (2011). Child health, developmental plasticity, and epigenetic programming. Endocr Rev.

[19] Vaiserman AM (2014). Early-life nutritional programming of longevity. J Dev Orig Health Dis.

[20] Buescher JL, Musselman LP, Wilson CA, Lang T, Keleher M, Baranski TJ, Duncan JG (2013). Evidence for transgenerational metabolic programming in Drosophila. Dis Model Mech.

[21] Somer RA, Thummel CS (2014). Epigenetic inheritance of metabolic state. Curr Opin Genet Dev.

[22] Na J, Musselman LP, Pendse J, Baranski TJ, Bodmer R, Ocorr K, Cagan R (2013). A Drosophila model of high sugar diet-induced cardiomyopathy. PLoS Genet.

[23] Bygren LO, Kaati G, Edvinsson S (2001). Longevity determined by paternal ancestors' nutrition during their slow growth period. Acta Biotheor.

[24] Chen KF, Crowther DC (2012). Functional genomics in Drosophila models of human disease. Brief Funct Genomics.

[25] Konsolaki M (2013). Fruitful research: drug target discovery for neurodegenerative diseases in Drosophila. Expert Opin Drug Discov.

[26] Pandey UB, Nichols CD (2011). Human disease models in Drosophila melanogaster and the role of the fly in therapeutic drug discovery. Pharmacol Rev.

[27] Guo A, Li L, Xia SZ, Feng CH, Wolf R, Heisenberg M (1996). Conditioned visual flight orientation in Drosophila: dependence on age, practice, and diet. Learn Mem.

[28] Xia SZ, Liu L, Feng CH, Guo AK (1997). Nutritional effects on operant visual learning in Drosophila melanogaster. Physiol Behav.

[29] Lee KP, Simpson SJ, Clissold FJ, Brooks R, Ballard JW, Taylor PW, Soran N, Raubenheimer D (2008). Lifespan and reproduction in Drosophila: New insights from nutritional geometry. Proc Natl Acad Sci U S A.

[30] Lee KP (2015). Dietary protein:carbohydrate balance is a critical modulator of lifespan and reproduction in Drosophila melanogaster: a test using a chemically defined diet. J Insect Physiol.

[31] Partridge L, Gems D, Withers DJ (2005). Sex and death: what is the connection?. Cell.

[32] Patel MS, Srinivasan M (2011). Metabolic programming in the immediate postnatal life. Ann Nutr Metab.

[33] Habbout A, Li N, Rochette L, Vergely C (2013). Postnatal overfeeding in rodents by litter size reduction induces major short- and long-term pathophysiological consequences. J Nutr.

[34] Raychaudhuri N, Thamotharan S, Srinivasan M, Mahmood S, Patel MS, Devaskar SU (2014). Postnatal exposure to a high-carbohydrate diet interferes epigenetically with thyroid hormone receptor induction of the adult male rat skeletal muscle glucose transporter isoform 4 expression. J Nutr Biochem.

[35] Gardebjer EM, Anderson ST, Pantaleon M, Wlodek ME, Moritz KM (2015). Maternal alcohol intake around the time of conception causes glucose intolerance and insulin insensitivity in rat offspring, which is exacerbated by a postnatal high-fat diet. FASEB J.

[36] Technau GM (1984). Fiber number in the mushroom bodies of adult Drosophila melanogaster depends on age, sex and experience. J Neurogenet.

[37] Sinakevitch I, Grau Y, Strausfeld NJ, Birman S (2010). Dynamics of glutamatergic signaling in the mushroom body of young adult Drosophila. Neural Dev.

[38] Barth M, Hirsch HV, Meinertzhagen IA, Heisenberg M (1997). Experience-dependent developmental plasticity in the optic lobe of Drosophila melanogaster. J Neurosci.

[39] Martino D, Loke YJ, Gordon L, Ollikainen M, Cruickshank MN, Saffery R, Craig JM (2013). Longitudinal, genome-scale analysis of DNA methylation in twins from birth to 18 months of age reveals rapid epigenetic change in early life and pair-specific effects of discordance. Genome Biol.

[40] Heisenberg M, Heusipp M, Wanke C (1995). Structural plasticity in the Drosophila brain. J Neurosci.

[41] Koyama T, Mendes CC, Mirth CK (2013). Mechanisms regulating nutrition-dependent developmental plasticity through organ-specific effects in insects. Front Physiol.

[42] Carone BR, Fauquier L, Habib N, Shea JM, Hart CE, Li R, Bock C, Li C, Gu H, Zamore PD, Meissner A, Weng Z, Hofmann HA (2010). Paternally induced transgenerational environmental reprogramming of metabolic gene expression in mammals. Cell.

[43] Fullston T, Palmer NO, Owens JA, Mitchell M, Bakos HW, Lane M (2012). Diet-induced paternal obesity in the absence of diabetes diminishes the reproductive health of two subsequent generations of mice. Hum Reprod.

[44] Pentinat T, Ramon-Krauel M, Cebria J, Diaz R, Jimenez-Chillaron JC (2010). Transgenerational inheritance of glucose intolerance in a mouse model of neonatal overnutrition. Endocrinology.

[45] Ding GL, Wang FF, Shu J, Tian S, Jiang Y, Zhang D, Wang N, Luo Q, Zhang Y, Jin F, Leung PC, Sheng JZ, Huang HF (2012). Transgenerational glucose intolerance with Igf2/H19 epigenetic alterations in mouse islet induced by intrauterine hyperglycemia. Diabetes.

[46] Jimenez-Chillaron JC, Isganaitis E, Charalambous M, Gesta S, Pentinat-Pelegrin T, Faucette RR, Otis JP, Chow A, Diaz R, Ferguson-Smith A, Patti ME (2009). Intergenerational transmission of glucose intolerance and obesity by in utero undernutrition in mice. Diabetes.

[47] Banerjee KK, Ayyub C, Ali SZ, Mandot V, Prasad NG, Kolthur-Seetharam U (2012). dSir2 in the adult fat body, but not in muscles, regulates life span in a diet-dependent manner. Cell Rep.

[48] Canto C, Auwerx J (2009). Caloric restriction, SIRT1 and longevity. Trends Endocrinol Metab.

[49] Clancy DJ, Gems D, Hafen E, Leevers SJ, Partridge L (2002). Dietary restriction in long-lived dwarf flies. Science.

[50] Fontana L, Partridge L (2015). Promoting health and longevity through diet: from model organisms to humans. Cell.

[51] Grandison RC, Piper MD, Partridge L (2009). Amino-acid imbalance explains extension of lifespan by dietary restriction in Drosophila. Nature.

[52] Matzkin LM, Johnson S, Paight C, Markow TA (2013). Preadult parental diet affects offspring development and metabolism in Drosophila melanogaster. PLoS One.

[53] Mirzaei H, Suarez JA, Longo VD (2014). Protein and amino acid restriction, aging and disease: from yeast to humans. Trends Endocrinol Metab.

[54] Musselman LP, Fink JL, Narzinski K, Ramachandran PV, Hathiramani SS, Cagan RL, Baranski TJ (2011). A high-sugar diet produces obesity and insulin resistance in wild-type Drosophila. Dis Model Mech.

[55] Pasco MY, Leopold P (2012). High sugar-induced insulin resistance in Drosophila relies on the lipocalin Neural Lazarillo. PLoS One.

[56] Zid BM, Rogers AN, Katewa SD, Vargas MA, Kolipinski MC, Lu TA, Benzer S, Kapahi P (2009). 4E-BP extends lifespan upon dietary restriction by enhancing mitochondrial activity in Drosophila. Cell.

[57] Allis D, Jenuwein T, Reinberg D (2007). Epigenetics.

[58] Lorbeck MT, Singh N, Zervos A, Dhatta M, Lapchenko M, Yang C, Elefant F (2010). The histone demethylase Dmel\Kdm4A controls genes required for life span and male-specific sex determination in Drosophila. Gene.

[59] Peleg S, Feller C, Forne I, Schiller E, Sevin DC, Schauer T, Regnard C, Straub T, Prestel M, Klima C, Schmitt Nogueira M, Becker L, Klopstock T (2016). Life span extension by targeting a link between metabolism and histone acetylation in Drosophila. EMBO Rep.

[60] Siebold AP, Banerjee R, Tie F, Kiss DL, Moskowitz J, Harte PJ (2010). Polycomb Repressive Complex 2 and Trithorax modulate Drosophila longevity and stress resistance. Proc Natl Acad Sci U S A.

[61] Smith EM, Hoi JT, Eissenberg JC, Shoemaker JD, Neckameyer WS, Ilvarsonn AM, Harshman LG, Schlegel VL, Zempleni J (2007). Feeding Drosophila a biotin-deficient diet for multiple generations increases stress resistance and lifespan and alters gene expression and histone biotinylation patterns. J Nutr.

[62] Esslinger SM, Schwalb B, Helfer S, Michalik KM, Witte H, Maier KC, Martin D, Michalke B, Tresch A, Cramer P, Forstemann K (2013). Drosophila miR-277 controls branched-chain amino acid catabolism and affects lifespan. RNA Biol.

[63] Vilmos P, Bujna A, Szuperak M, Havelda Z, Varallyay E, Szabad J, Kucerova L, Somogyi K, Kristo I, Lukacsovich T, Jankovics F, Henn L, Erdelyi M (2013). Viability, longevity, and egg production of Drosophila melanogaster are regulated by the miR-282 microRNA. Genetics.

[64] Dunwell TL, Pfeifer GP (2014). Drosophila genomic methylation: new evidence and new questions. Epigenomics.

[65] Capuano F, Mulleder M, Kok R, Blom HJ, Ralser M (2014). Cytosine DNA methylation is found in Drosophila melanogaster but absent in Saccharomyces cerevisiae, Schizosaccharomyces pombe, and other yeast species. Anal Chem.

[66] Panikar CS, Rajpathak SN, Abhyankar V, Deshmukh S, Deobagkar DD (2015). Presence of DNA methyltransferase activity and CpC methylation in Drosophila melanogaster. Mol Biol Rep.

[67] Radford EJ, Ito M, Shi H, Corish JA, Yamazawa K, Isganaitis E, Seisenberger S, Hore TA, Reik W, Erkek S, Peters AH, Patti ME, Ferguson-Smith AC (2014). In utero effects. In utero undernourishment perturbs the adult sperm methylome and intergenerational metabolism. Science.

[68] Zhao HW, Haddad GG (2011). Review: Hypoxic and oxidative stress resistance in Drosophila melanogaster. Placenta.

[69] Guerrero-Bosagna C, Jensen P (2015). Globalization, climate change, and transgenerational epigenetic inheritance: will our descendants be at risk?. Clin Epigenetics.

[70] Pembrey M, Saffery R, Bygren LO (2014). Human transgenerational responses to early-life experience: potential impact on development, health and biomedical research. J Med Genet.

