# Supplementary material for: Transgenerational programming of longevity and reproduction by post-eclosion dietary manipulation in Drosophila
Source: Aging (Albany NY). 2016 Mar 28;8(5):1115–29. doi: 10.18632/aging.100932 (PMC4931857; doi:10.18632/aging.100932)
Supplement: Supplementary file 1 [file aging-08-1115-s001.pdf]

SUPPLEMENTAL FIGURES

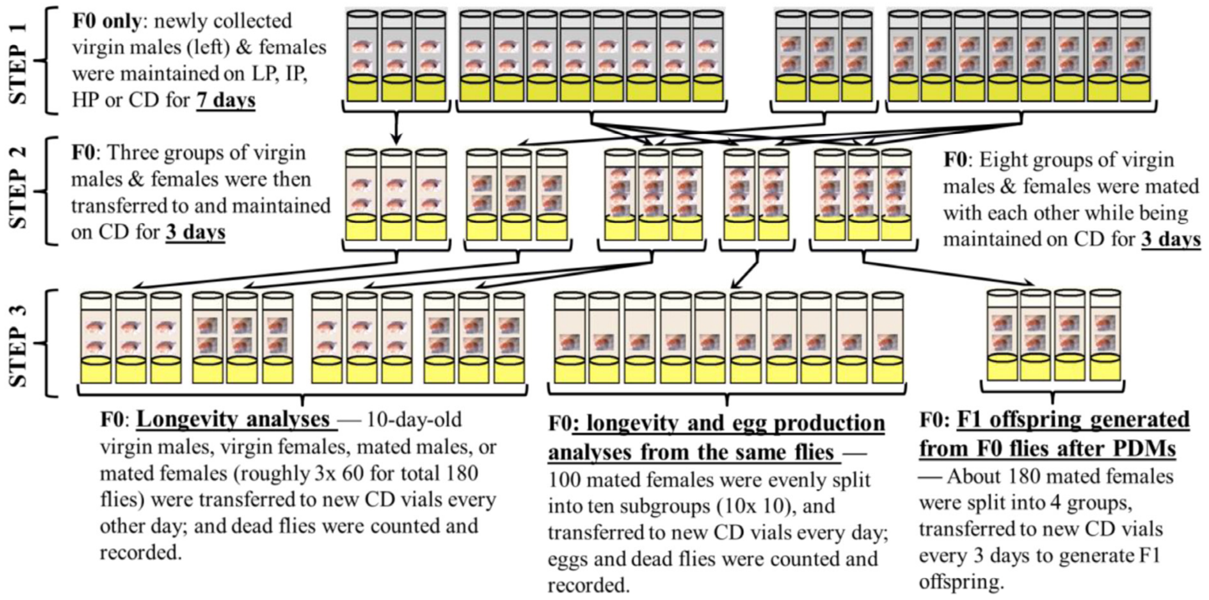

Supplemental Figure S1. Experimental design and procedures. Shown here is the F0 generation, subjected to the 7-day PDMs before longevity and fecundity analyses. **STEP 1**) Virgin males and females were collected and subjected to 7-day PDMs. **STEP 2**) Three groups of 60 virgin males and females were transferred to CD for 3 days, while the others (8 groups) were mated with each other for 3 days on CD. **STEP 3**) *Longevity analyses:* three groups (~180 flies) of virgin males, virgin females, mated males, and mated females were used; *egg production and longevity analyses from same flies:* 100 mated females were evenly split into 10 subgroups (10x10) and used for analyses; *F1 offspring generated from the F0 flies after PDMs:* 180 mated females were split into 4 groups and used for generating the F1 offspring while being maintained on CD all the time. Similar analyses were done with their F1, F2, and F3 offspring, by repeating **STEPs 2–3** (i.e., without PDMs from **STEP 1**) as for the F0 parents while using newly-born virgin males and females, except that roughly 4x 50 flies were used for longevity analyses of the F2–F3 offspring.

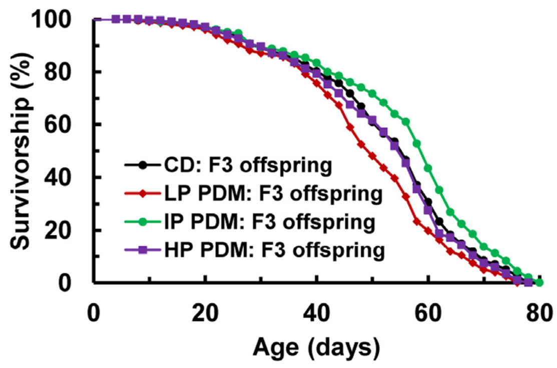

Supplemental Figure S2. Survival curves for the F3 generation (mated females only). The LP PDM of the F0 flies shortened longevity of their F3 offspring significantly (P = 0.003; Mantel-Cox test), or by 11% at the median lifespan (50 vs. 56 for LP vs. CD); while the IP PDM of the F0 flies improved longevity of their F3 offspring (P = 0.005), or 7% at the median lifespan (60 vs. 56 for IP vs. CD). The HP PDM of F0 flies induced no effect on F3 offspring any further (P = 0.46; 56 vs. 56 for median lifespan).

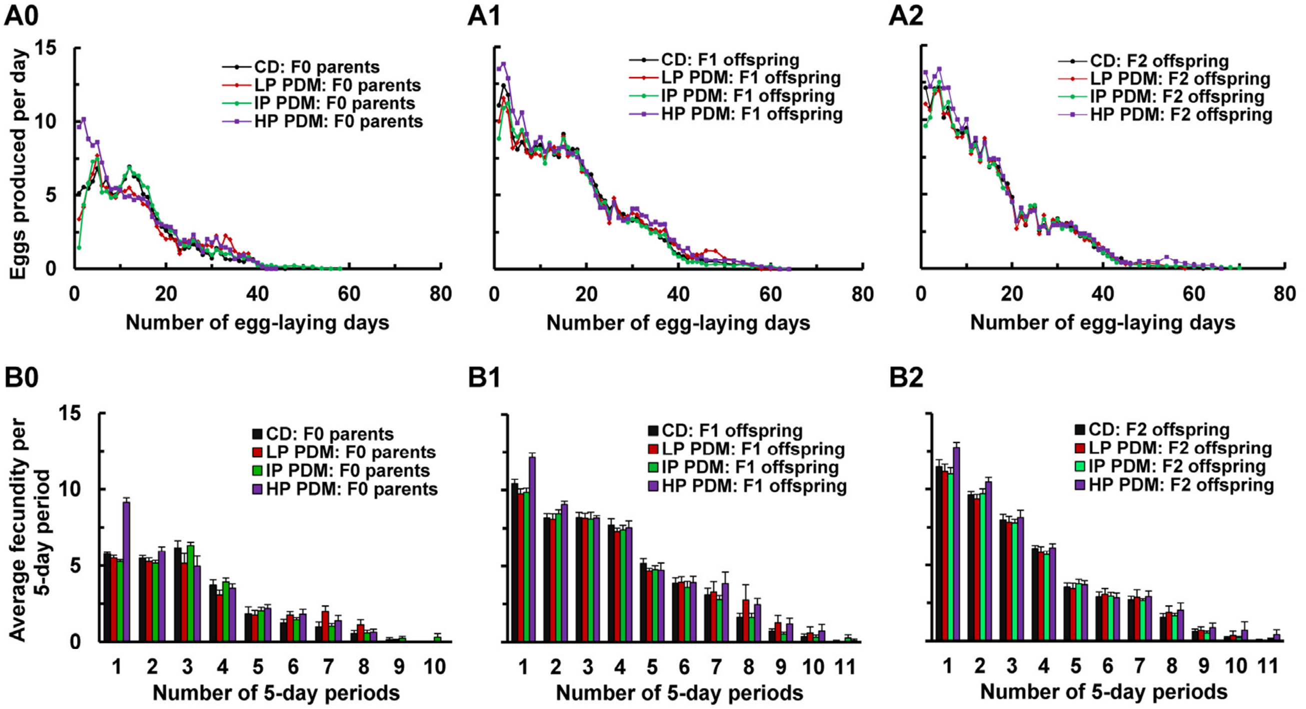

Supplemental Figure S3. Eggs produced per day and 5-day period. (**A**) Average number of "*Eggs Produced per Day*" (1st row) and (**B**) "*Fecundity per 5-day Period*" (2nd row); and (**0**) F0 parents (1st column), (**1**) F1 offspring (2nd column), and (**2**) F2 offspring (3rd column) by the 100 mated females. "Eggs Produced per Day" was calculated as "total eggs produced for a given day divided by the number (1–100) of the surviving flies within a diet group". "Fecundity per 5-day Period" was defined as the average number of eggs laid over a 5-day period by one mated female.

SUPPLEMENTAL TABLES

Supplemental Table S1. Diets used for the PDMs of the F0 parents.

	Ingredients	Control diet (CD) [A]	LP diet [B]	IP diet [C]	HP diet [D]
Recipes	Yellow cornmeal (gm)	76.6	98.0	92.0	162.5
	Yeast (gm)	32.1		17.2	33.5
	Agar (gm)	9.3	10.0	5.2	7.5
	Glucose (gm)	63.2	150.0	147.4	
	Sucrose (gm)	31.6			40.0
	CaCl2 (gm)	0.7			
	Soy flour (gm)				30.0
	Water (gm)	1000	1000	1000	1000
**Total (gm, with water)**		1213.5	1258.0	1261.8	1273.5
**Protein (%; with water)** [E]		8.6	3.3	5.5	13.5
**Carbohydrate (%; with water)** [E]		76.6	90.5	87.4	69.6
**Total calories** [E]		758.0	969.4	973.6	982.6
**Calories per gram** [F]		0.62	**0.77**	**0.77**	**0.77**

Provided are the recipes, along with the protein, carbohydrate, and calorie information for the control diet (CD) and 3 other diets used for the PDMs of the F0 parents. (**A**) CD is a food medium routinely used in the lab. (**B**) The "LP" (Low Protein) diet was adapted from Xia et al [28]. (**C**) The "IP" (Intermediate Protein) diet was adapted from Guo et al [27]. (**D**) The "HP" (High Protein) diet was adapted from a widely used "standard diet" as described by Guo et al [27] and at Bloomington Stock Center (http://flystocks.bio.indiana.edu/Fly_Work/media-recipes/bloomfood.htm). (**E**) The protein, carbohydrate, and calorie information for all the ingredients has been obtained from their labels or from http://nutritiondata.self.com/. (**F**) All three diet recipes were slightly modified to be isocaloric (0.77 calories/gm food).

Supplemental Table S2. Sample size (N) for four types of flies after each PDM across the F0–F3 generations in the longevity experiments.

	PDM diets	F0 parents (+ PDM)	F1 offspring	F2 offspring	F3 offspring
**Virgin males**	CD (control)	181	184	198	–
	LP (F0 only)	183	182	202	–
	IP (F0 only)	180	181	200	–
	HP (F0 only)	182	180	201	–
**Virgin females**	CD (control)	180	183	201	–
	LP (F0 only)	182	182	202	–
	IP (F0 only)	184	178	203	–
	HP (F0 only)	180	185	201	–
**Mated males**	CD (control)	181	180	199	–
	LP (F0 only)	182	181	200	–
	IP (F0 only)	179	181	201	–
	HP (F0 only)	180	185	202	–
**Mated females**	CD (control)	185	183	199	202
	LP (F0 only)	183	181	203	202
	IP (F0 only)	182	183	200	205
	HP (F0 only)	181	182	201	203
**Mated females (**same flies also assayed for reproduction**)**	CD (control)	100	100	100	–
	LP (F0 only)	100	100	100	–
	IP (F0 only)	100	100	100	–
	HP (F0 only)	100	100	100	–
**Subtotal (**per generation**)**		**3305**	**3311**	**3613**	**812**
**Total (**whole study**)**			**11,041**		

Supplemental Table S3. Number of eggs recorded for each subgroup (10 flies) of the mated females from the F0 parents (with PDM) and their F1-F2 offspring.

PDM diets			F0 parents (+ PDM)	F1 offspring	F2 offspring
**Mated females** (10x subgroups of 10 flies; same flies also assayed for longevity)	**CD** (control)	Subgroup 1	1101	2037	2022
		Subgroup 2	1139	2057	1961
		Subgroup 3	1070	2072	2095
		Subgroup 4	1192	2111	1982
		Subgroup 5	1213	2040	1987
		Subgroup 6	1088	2253	2060
		Subgroup 7	1122	2096	2027
		Subgroup 8	1034	2132	2096
		Subgroup 9	1017	2123	2061
		Subgroup 10	957	2160	2118
		Mean ± SEM	1093.3 ± 24.8	2108.1 ± 20.6	2040.9 ± 17.0
		Total per generation	10933	21081	20409
	**LP** (F0 only)	Subgroup 1	916	2008	1854
		Subgroup 2	902	1950	1796
		Subgroup 3	769	1794	1926
		Subgroup 4	899	1946	1947
		Subgroup 5	1015	1853	1811
		Subgroup 6	825	1891	1923
		Subgroup 7	873	1719	1867
		Subgroup 8	902	1996	1704
		Subgroup 9	834	1859	1908
		Subgroup 10	1014	1716	1731
		Mean ± SEM	894.9 ± 24.5	1873.2 ± 33.4	1846.7 ± 26.7
		Total per generation	8949	18732	18467
	**IP** (F0 only)	Subgroup 1	1095	2150	2128
		Subgroup 2	1287	2315	2165
		Subgroup 3	1100	2252	2115
		Subgroup 4	1127	2176	2089
		Subgroup 5	1336	2205	2152
		Subgroup 6	1187	2348	2196
		Subgroup 7	1074	2168	2199
		Subgroup 8	1242	2222	2161
		Subgroup 9	1161	2062	1942
		Subgroup 10	1237	2211	2213
		Mean ± SEM	1184.6 ± 28.0	2210.9 ± 25.9	2136.0 ± 24.8
		Total per generation	11846	22109	21360
	**HP** (F0 only)	Subgroup 1	1061	2288	2154
		Subgroup 2	1214	2148	2163
		Subgroup 3	1180	2168	2130
		Subgroup 4	1083	2163	2256
		Subgroup 5	1321	2199	2177
		Subgroup 6	1140	2292	1952
		Subgroup 7	1121	2132	2183
		Subgroup 8	1217	2172	2052
		Subgroup 9	1298	2076	2057
		Subgroup 10	1100	2257	1992
		Mean ± SEM	1173.5 ± 28.1	2189.5 ± 22.2	2111.6 ± 30.1
		Total per generation	11735	21895	21116
**Subtotal** (per generation across four diets)			**43463**	**83817**	**81352**
**Total** (whole study)				**208,632**	
